# Syndromic Surveillance in Tribal Health: Perspectives from Three Tribal Epidemiology Centers on Access and Utilization

**DOI:** 10.3390/ijerph22050664

**Published:** 2025-04-23

**Authors:** Cheng Wang, Lowrie Ward, Nicole Holdaway Smith

**Affiliations:** 1 Great Plains Tribal Leaders’ Health Board, Rapid City, SD 57703, USA; 2 Alaska Native Tribal Health Consortium, Anchorage, AK 99508, USA; laward@anthc.org; 3 Northwest Portland Area Indian Health Board, Portland, OR 97201, USA; nsmith@npaihb.org

**Keywords:** syndromic surveillance, tribal health, public health authority

## Abstract

Syndromic surveillance has evolved into a vital public health tool, providing near real-time data to detect and respond to health threats. While states administer syndromic surveillance systems, Tribal Epidemiology Centers (TECs) serve American Indian and Alaska Native (AIAN) communities across multistate regions, often encountering significant barriers to data access and utilization. This manuscript explores how TECs access and use syndromic surveillance data to address health disparities in AIAN populations, highlighting successes, innovations, and ongoing challenges. The Alaska Native Epidemiology Center (ANEC), Great Plains Tribal Epidemiology Center (GPTEC), and Northwest Tribal Epidemiology Center (NWTEC) provide insights into their syndromic surveillance practices. This includes data access methods, the creation of dashboards and reports, technical assistance for Tribal Health Organizations (THOs), and strategies for overcoming jurisdictional and data-sharing barriers. TECs have successfully leveraged syndromic surveillance to monitor critical health issues, including respiratory illnesses, substance misuse, behavioral health, and maternal care. Collaborative efforts have addressed race misclassification and data gaps, enabling targeted interventions such as air purifier distribution and improving health care delivery for tribal veterans. However, TECs can face restrictive data use agreements, jurisdictional misalignments, and limited access to granular data, hindering their ability to serve AIAN communities comprehensively. Syndromic surveillance offers transformative potential for improving public health in AIAN communities. To fully realize this potential, systemic changes are needed to streamline data-sharing agreements and improve data accuracy. These efforts, along with strong collaborations between TECs and state health departments, are critical to advancing health equity, respecting tribal sovereignty, and ensuring timely, actionable insights for AIAN populations.

## 1. Introduction

The National Syndromic Surveillance Program (NSSP), administered by the Centers for Disease Control and Prevention (CDC), collects and uses a wide array of health-related data for public health surveillance. The system was originally created for the early detection of health threats and has become a vital tool for states and localities to detect illnesses and biological agents [1]. Data sources included in the NSSP come from various healthcare settings such as emergency departments (EDs), urgent care centers, inpatient visits, laboratory data, ambulatory care visits, pharmacy data, school absenteeism reports, and Emergency Medical System (EMS) data. These data sources allow for near real-time monitoring of disease outbreaks and other public health emergencies. Today, many states participate in the CDC’s NSSP, using the BioSense Platform [2] to track a broad range of health events. Other states use similar platforms with secure data protocols.

The scope of syndromic surveillance has expanded beyond infectious disease to include behavioral health, air quality-related illnesses, injuries, and more. A national Community of Practice has fostered idea-sharing and support, resulting in an increased number of nationally validated syndrome definitions. Research has shown that syndromic surveillance can provide important information about suicidal ideation [3], disaster response [4], and firearm injuries [5], among others.

Despite syndromic surveillance expanding beyond its original intended use in public health, data access and administration methods have remained unchanged. Medical facilities that submit data to syndromic surveillance systems are the data owners. States act as administrators for the data of facilities in their jurisdiction, with some participating in the CDC’s NSSP Biosense Platform, a secure, cloud-based electronic health information system, while some states administer their own data platforms. Many states have enacted laws or regulations requiring hospitals, emergency departments, and other healthcare facilities to submit patient visit data to centralized syndromic surveillance systems. In some jurisdictions, the requirements are mandatory and enforced through public health codes, while other states provide incentives or guidance for facilities to participate voluntarily. As part of this process, states often establish formal data use agreements (DUAs) with the reporting facilities, which outline what data are collected, how they are shared, and the conditions under which external entities may be granted access. While these DUAs safeguard patient confidentiality and ensure compliance with privacy laws, they can also place restrictions on data sharing, limiting the level of detail or timeliness available to organizations outside of the state health department.

**Figure 1:**
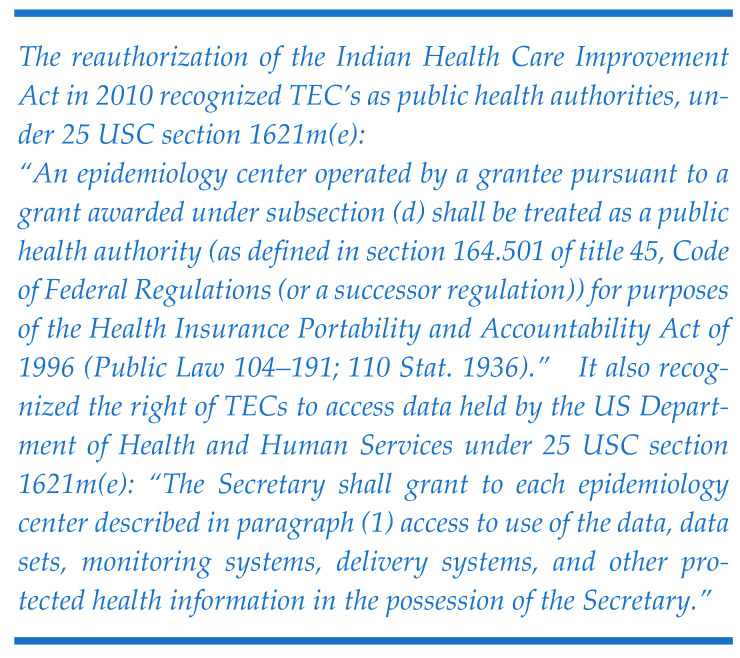
Excerpt from the law recognizing the public health authority of TECs. Reprinted from refs. [7,8].

Tribal Epidemiology Centers (TECs), established in 1996 after the authorization of the Indian Health Care Improvement Act (IHCIA), work with tribes, tribal, and urban Indian health organizations to collect health status data, evaluate and monitor health delivery systems, identify local priorities for health care delivery, and implement culturally appropriate public health practice for American Indian and Alaska Native (AIAN) communities [6]. Despite all twelve TECs being recognized as public health authorities (PHAs) since 2010 and having the right to access data held by the US Department of Health and Human Services (HHS) for various public health activities [Figure 1] [7,8], TECs must work with state health departments to receive syndromic surveillance data through the syndromic surveillance platforms available in their jurisdictions. This can pose challenges since tribal nations may intersect multiple state boundaries, and many TECs serve tribal organizations across multiple states, resulting in inconsistent data access. Moreover, not all states recognize the PHA status of TECs [8] or share syndromic data with them. This lack of common understanding and established standard practices makes it difficult for TECs to access these crucial data.

Educating state health departments and ensuring that both parties, including both legal teams, are aligned often requires significant time and effort, yet it remains essential for enabling TECs to access the crucial syndromic data needed to serve their communities effectively. This manuscript will explore how three TECs obtain and utilize syndromic surveillance data to support their partner tribes and Tribal Health Organizations (THOs). We will highlight innovations and success stories and advocate for a more streamlined approach for TECs to access syndromic surveillance data to support their critical work. The goal is to offer practical, actionable insights for other tribes and tribal organizations seeking access to this system.

### TEC Descriptions

The Alaska Native Epidemiology Center (ANEC) is located within the Alaska Native Tribal Health Consortium’s (ANTHC) Community Health Services division. ANTHC, along with Alaska’s regional THOs, comprise the Alaska Tribal Health System, which has the primary goal of improving the health of Alaska Native People. As of 2023, Alaska has 150,374 Alaska Native and American Indian residents and 160,646 Indian Health Service users [9]. Most THOs have facilities that participate in the state’s syndromic program.

The Great Plains Tribal Epidemiology Center (GPTEC), a division of the Great Plains Tribal Leaders’ Health Board, serves 18 tribal communities in North Dakota (ND), South Dakota (SD), Iowa (IA), and Nebraska (NE). GPTEC’s mission is to improve health outcomes and eliminate the health disparities for AIAN people in the area. In the GPA area, the AIAN population is served by both private hospitals and IHS facilities. However, IHS facilities do not submit data to the state-run syndromic system. Among the four states in our area (SD, ND, IA, and NE), only the South Dakota Health Department provides syndromic data access through the ESSENCE platform. Nebraska, on the other hand, shares a weekly spreadsheet with aggregated data on selected topics. As a result, only the AIAN population utilizing private hospital emergency departments in South Dakota are included in the ESSENCE syndromic data system shared with the GPTEC.

The Northwest Tribal Epidemiology Center (NWTEC), a division of the Northwest Portland Area Indian Health Board (NPAIHB), serves the 43 federally recognized tribes of Idaho, Oregon, and Washington State, with the goal of supporting tribes to improve their health and quality of life. As the area has no Indian Health Service or tribal hospitals, the AIAN population is served by community hospitals and members are expected to be represented in syndromic data.

## 2. Accessing and Utilizing Syndromic Data: Current Approaches and Impacts

### 2.1. Access

TECs employ diverse methods to access and analyze syndromic surveillance data due to jurisdictional differences and state-specific policies and practices. These methods are shaped by existing syndromic data infrastructure, DUAs, system permissions, and the resources available within each TEC’s service area. The following describes general approaches and processes used by TECs while acknowledging variability in state practices.

TECs typically access syndromic surveillance data through state-run software systems or the national ESSENCE system. Access begins with negotiating permissions and conditions for data use with state administrators. This process is typically time-consuming and must be handled cautiously to secure a written and signed DUA. Alternatively, some TECs, such as the GPTEC, access syndromic data through a mutual understanding and oral agreement with state health departments, without requiring a DUA, recognizing their roles as PHAs. These agreements dictate whether TECs can access aggregate or line-level data and may impose additional restrictions, such as limiting data-sharing or requiring prior approval for analyses and publications. User permissions within the systems are assigned by state administrators. Typically, access to syndromic surveillance systems is provided via username and password, with permissions assigned by state administrators. In some cases, TECs are granted the same permissions as state epidemiologists, enabling full access to line-level data for all visits to any reporting facility in the state. However, in many instances, access is restricted to aggregate reports or limited to tribal facilities that explicitly grant permission. Refer to Table 1 for detailed information on the state-by-state overview of TECs’ syndromic system access, types of access, and DUA formats and requirements.

### 2.2. Use

Once access is granted, TECs interact with the data in several ways:

- Querying and Filtering: TECs use built-in tools and customized queries within syndromic systems to filter data by parameters such as geographic region, facility type, and specific health conditions.
- Combining Data Sources: To address gaps in single-state data and align with tribal jurisdictions, TECs may combine data from multiple systems. For example, data may be aggregated by Purchased/Referred Care Delivery Area (PRCDA) counties, which may span state lines.
- Data Integration: Some TECs create Application Program Interfaces (APIs) to automate data retrieval, while others manually download data from individual systems, ensuring compliance with the conditions of individual DUAs by keeping datasets separate when required.

Processed data are transformed into user-friendly formats for tribal and partner use. TECs create fact sheets, dashboards, charts, and alerts tailored to the needs of THOs and communities. Specific examples include the following:

- Dashboards: Visualizing trends in ED visits for specific conditions, such as respiratory illnesses.
- Alerts: Monitoring system-defined thresholds to notify tribes of unusual trends or emerging public health concerns.
- Reports: Preparing regular updates summarizing key findings for tribal governments, health departments, and other stakeholders.

### 2.3. Impacts

TECs, including the ANEC, GPTEC, and NWTEC, use syndromic surveillance systems to understand and address the healthcare needs of AIAN communities across diverse regions. Each center utilizes syndromic data platforms, provides technical assistance to THOs, and shares surveillance findings with healthcare providers and public health partners to improve health outcomes in tribal communities.

**Figure 2:**
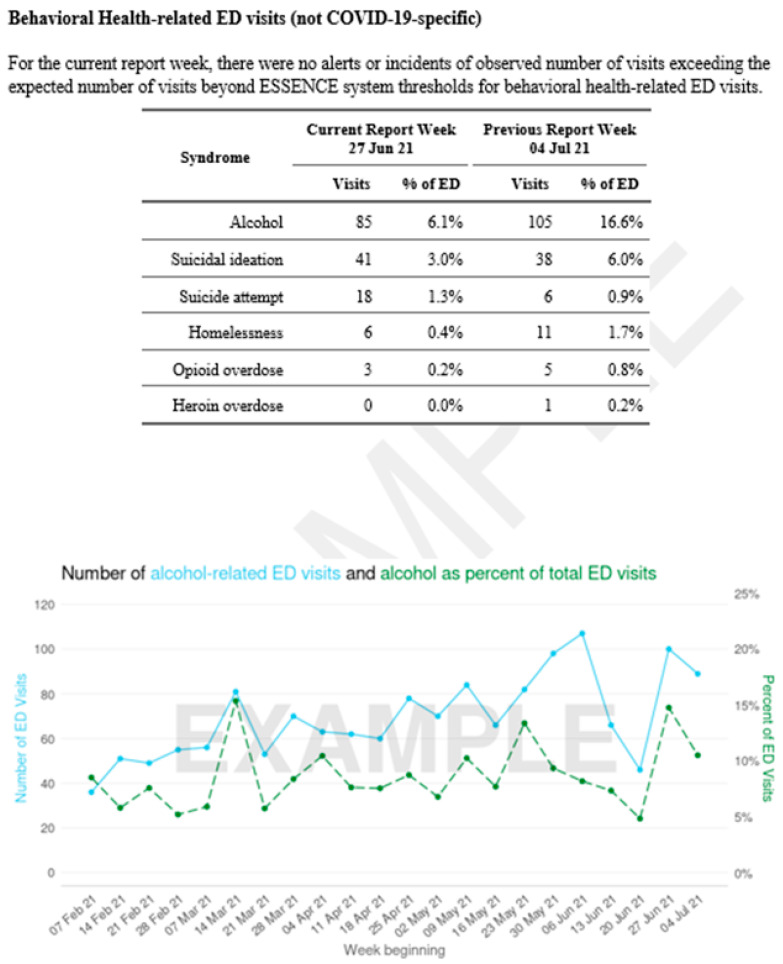
Example of a report (using fictional data) created by the ANEC for Tribal Health Organizations that participate in syndromic surveillance. Reports contain tables and figures generated for syndromic surveillance facilities that are interested in tracking.

The ANEC uses ESSENCE to create several dashboards specific to facilities or geographic areas and uses R/R Markdown to build regularly updated reports tailored to each THO partner’s needs [Figure 2]. When potential concerns are identified in the data, ANEC alerts the facilities via email, although formal alerts are issued by the Alaska Department of Health. The ANEC supports six THOs, covering seven emergency departments and two regional clinic networks, and has provided numerous surveillance reports to these facilities. During the height of the COVID-19 pandemic (2020–2022), the ANEC shared weekly reports to keep physicians updated on COVID-related visits and behavioral health concerns. Additional data tracking included syndromic trends associated with significant events like Typhoon Merbok, drug overdose clusters, and alcohol-related visits, providing THOs with actionable insights for patient care and emergency response. The ANEC has advocated for and supported tribal hospital inclusion in the ESSENCE platform. The inclusion of THO facilities in the system allows for a more accurate representation of AIAN and rural residents in Alaska’s syndromic surveillance.

The GPTEC utilizes the ESSENCE platform to develop dashboards that monitor critical health conditions, such as substance misuse, through multiple panels that enable data comparison across age, gender, and geography. These dashboards serve as valuable tools to track health trends and respond to specific data requests from tribal communities. In response to the unique challenges faced by tribal veterans, the GPTEC has also partnered with researchers to study how physical distance from VA facilities affects veteran health outcomes in tribal areas. Findings from this research are intended to inform healthcare delivery improvements for tribal veterans. The GPTEC has actively presented its syndromic surveillance methods at national conferences, sharing insights on data access, utilization, and public health applications to empower other tribal organizations.

The NWTEC has leveraged syndromic data to understand and address the healthcare burdens faced by AIAN communities, particularly in areas such as behavioral health, overdoses, extreme weather-related health outcomes, maternal care, and respiratory illness. By conducting in-depth case reviews on specific syndromes—such as incidents involving unhoused individuals, pedestrian-versus-auto accidents, and injuries associated with commercial fishing—the NWTEC has been able to provide tailored insights that guide program planning and evaluation. These reviews have led to targeted efforts, like adapting first aid and safety training for tribal commercial fishers and distributing air purifiers to areas with high rates of air quality-related emergency department visits. Tracking these visits over time allows for evaluation of these interventions’ impact on community health outcomes.

From 2020 to mid-2022, the NWTEC published a public-facing dashboard that tracked COVID-19, influenza, mental health emergencies, vaccine-related adverse events, and other high-priority conditions across AIAN and non-AIAN populations. This dashboard, updated and shared with tribal leaders and public health officials at weekly meetings, provided critical insights for timely public health responses.

**Figure 3:**
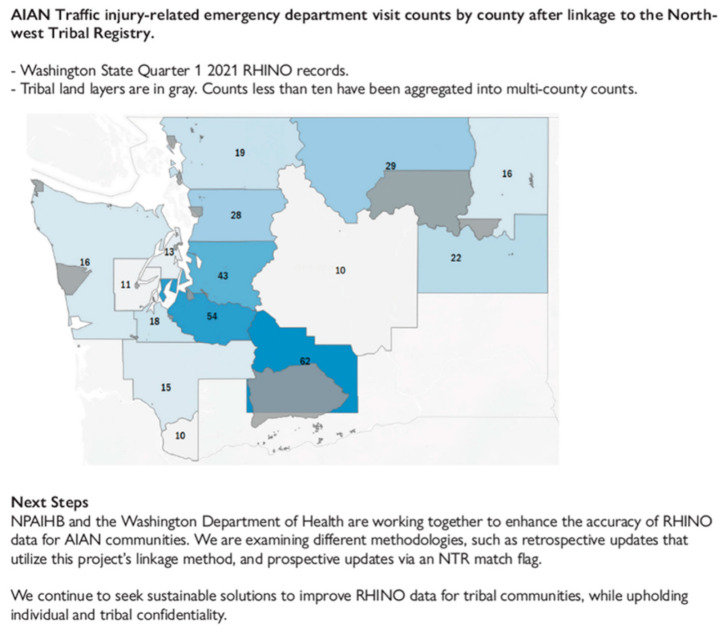
Example of a newsletter article published through the NWTEC and Washington Department of Health’s partnership to enhance the accuracy of syndromic data for AIAN communities. Reprinted from Tom Hulse, Meena Patil, Nicole Smith and Sujata Joshi (2023).

Additionally, the NWTEC has partnered with the Washington Department of Health to address race misclassification in emergency department data. By linking data from Washington’s RHINO system with the Northwest Tribal Registry, the NWTEC identified significant underestimates in emergency care visit counts for AIAN populations—particularly for motor vehicle injuries, which were underreported by 45%. This finding has informed ongoing efforts to improve race data accuracy in the syndromic surveillance system, allowing for a more precise understanding of health outcomes among AIAN individuals [Figure 3]. Through these initiatives, the NWTEC continues to enhance the quality of health data for AIAN communities, leading to more effective public health efforts.

Through their respective syndromic surveillance efforts, the ANEC, GPTEC, and NWTEC have empowered tribes and THOs with timely health data, addressed specific tribal health needs, and fostered collaborations to improve data accuracy and accessibility. This work continues to strengthen public health responses and healthcare delivery in tribal communities, contributing to better health outcomes and equity for AIAN populations.

## 3. Discussion of Challenges Encountered

Legal, relational, and technical barriers are limiting TECs’ access to syndromic surveillance data. First, most state health departments failed to fully recognize their legal obligation to share data with tribes and tribal organizations. The IHCIA and its subsequent amendments affirmed that the federal government is responsible for ensuring “the highest possible health status for Indians and urban Indians and to provide all resources necessary to effect that policy” [10]. In the context of Indian affairs, federal law preempts state law [11], and these data are central for achieving effective tribal public health practice, which means states hold the legal obligation to share data with tribes and tribal organizations as well. Second, state health departments are at different stages in recognizing the PHA status of tribes and tribal organizations and acknowledging that TECs as PHAs can receive protected health information under the Privacy Rule of the Health Insurance Portability and Accountability Act (HIPAA) [12]. Third, many state health departments have yet to fully recognize the urgency and significance of sharing data with tribes and tribal organizations. Historically, these departments have functioned as the primary custodians and gatekeepers of data, which has led to the major emphasis on data security in the context of data sharing. However, the potential harm caused by withholding data, particularly to the health of tribal populations, is often overlooked. The COVID-19 pandemic highlighted this issue, as the life expectancy of the AIAN population declined more sharply than that of any other major ethnic group, underscoring the critical need for more collaborative data-sharing efforts. This disparity should serve as a call to action for state health departments to reconsider their approach to data sharing with tribal communities. Fourth, developing the relationship between TECs and state health departments, and fostering trust and collaboration, takes significant time, effort, and strategic planning, especially in an environment with high levels of historical disagreement and misunderstanding. Another significant challenge lies in the jurisdictional mismatch between states and TECs. Most TECs operate within regions defined by Indian Health Service Areas, which frequently span multiple states [6]. TECs must collaborate with several states to access data for their entire service area. However, each state has its own data-sharing regulations and varying recognition of TECs’ PHA. Some states do not provide any syndromic surveillance data access to their area TEC despite the PHA held by TECs. States with small AIAN populations can face statistical reliability issues when analyzing data in isolation, further complicating state data-sharing agreements, which may restrict sharing small numbers. Lastly, the absence of standardized guidelines or pathways, coupled with a lack of staff with the necessary technical skills, further complicates this process. Access to syndromic surveillance data for TECs varies significantly by jurisdiction. The state-administered nature of the systems means that access methods, permissions, and data-sharing protocols are inconsistent across states. Taken together, TECs gaining access to the syndromic surveillance system is an exceptionally complex process involving multiple stakeholders and sensitive issues.

In addition, for TECs that have gained access to syndromic surveillance data, the level of access to line-level data, personally identifiable information (PII), and comprehensive race and ethnicity data varies significantly.

Line-level data with details on individual patient encounters are essential for TECs to deliver useful and actionable data to the tribes and THOs they serve. However, access to such granular data varies widely. In the most restrictive cases, states provide solely aggregate data to TECs, which can be extremely limiting. Syndromic queries are based on symptoms, and line-level encounter details are essential for reviewing cases for further investigation [13]. Additionally, reviewing the encounter details and fine-tuning the queries to exclude false positives is a critical process when constructing a high-quality query [14,15]. Because data quality and clinical coding practices vary across states, this iterative query refinement process is vital for data accuracy and cannot be replaced by applying a query built by other states or entities.

PII is sometimes unavailable in syndromic systems, yet it is crucial for accurate data analysis [16]. PII allows epidemiologists to identify repeat encounters or multiple visits by the same individual. Without access to line-level data and unique patient identifiers, epidemiologists cannot distinguish between unique and repeat patients, making it difficult to accurately assess trends or anomalies. When facilities choose not to share PII or when state laws do not require or allow its reporting, all PHAs, including state health departments, face the same limitations. Racial misclassification of AIAN individuals in health care data is well documented [17]. Line-level data with PII are essential for correcting these errors, with which TECs and states can work together successfully and safely to link data and reduce misclassification.

In some cases, full population data with all race and ethnicity groups are not shared with TECs. AIAN people use both IHS and non-IHS facilities. Some states grant access at the facility level, when the facilities have approved or requested it, which can be effective when tribal entities own or operate the facilities. However, this approach excludes data from non-tribal facilities, creating gaps in data surveillance for AIAN people who visit non-tribal facilities. Additionally, most IHS facilities do not submit syndromic data into the state-run syndromic systems. Data from all AIAN health care visits are essential for understanding the burden of specific health conditions and describing health care utilization by AIAN. These fragmented data systems and jurisdictional disparities make it extremely difficult for TECs to achieve a comprehensive understanding of public health within their regions.

Furthermore, DUAs written by states may impose additional restrictions, such as requiring TECs to run analyses through state administrators prior to sharing with tribes or publication. This can limit the ability of TECs to share critical data with tribes–sovereign nations with the right to make informed decisions for their citizens. For instance, restrictions like prohibiting sharing counts under ten create barriers to transparency and data-driven decision-making. Requiring TECs to obtain state approvals before sharing data with tribes undermines tribal sovereignty and hinders TECs’ ability to fully leverage syndromic data to serve AIAN communities effectively [18].

While the challenges described in this paper have primarily focused on variability related to data access, it is important to acknowledge that variability exists extensively throughout syndromic surveillance systems nationwide. State requirements for facility reporting vary across the country, leading to inconsistencies in the data that are available to all PHAs. Reporting also varies based on the capabilities of each Electronic Medical Record system. Indian Health Service facilities do not participate in the National Syndromic Surveillance system, and the syndromic surveillance data they collect are not known to be available to TECs or state health departments. Additionally, technological issues further complicate data sharing. For example, many states may wish to share tribe-specific data with TECs, but the National ESSENCE system does not capture tribal affiliation. Similarly, states cannot grant line-level access to all visits by AIAN persons if the system’s access management does not offer that option.

## 4. Conclusions and Future Directions

Syndromic surveillance has become an invaluable tool for monitoring public health trends and responding to emerging health concerns. TECs have utilized real-time syndromic data to support tribes in public health action and in their delivery of high-quality health care. The successes highlighted in this paper underscore the potential impact of syndromic surveillance when TECs are empowered with the access they need. Significant barriers remain, including accessing data across jurisdictions, restrictive data use agreements, and misclassification issues, which limits the ability of TECs to fulfill their roles as PHAs and serve tribal nations and THOs effectively. To address these challenges, there is a need for a more streamlined and standardized approach to data access and data sharing that respects the sovereignty of tribes. Providing TECs with equitable access to granular data, in keeping with TECs’ PHA status, will ensure that TECs can collaborate with state health departments and tribal organizations to improve health and quality of life for AIAN people across the nation.

Ongoing advocacy for the legal obligation of tribal data access and recognition of tribal and tribal organizations’ PHA status are essential. These factors form the critical foundation for fostering interest in data-sharing and -securing agreements. Building upon these foundational elements, the development of standardized approaches and guidelines might become a viable next step.

Additionally, to improve TECs’ access to syndromic surveillance data, policy changes at both the federal and state levels are essential. TECs have advocated for this through consultations and engagements with federal agencies, such as the CDC Tribal Advisory Committee and the HHS Secretary’s Tribal Advisory Committee. In its 4 March 2022, report, the Government Accountability Office (GAO) recommended that the CDC develop clear guidance and procedures for TECs to request data, including details on available data, request processes, agency contacts, review criteria, and response timeframes. Additionally, TECs are working with states to update their tribal data-sharing policies. For instance, the GPTEC has actively provided feedback on the Iowa State Health Department’s tribal data-sharing policies, particularly during the recent policy review and update process. Consequently, the most recent revision of their tribal data-sharing policy acknowledges TECs’ PHA status and incorporates guidance on tribal data-sharing. While policies are evolving, they have not yet sufficiently simplified access to syndromic surveillance data for tribes and tribal organizations. Ongoing efforts are necessary to advocate for policy changes at both the federal and state levels.

## Figures and Tables

**Table 1:** State-by-state overview of TECs’ syndromic system access, type of access, and DUA format and requirements.

State	TEC Access Date	Type of Access	DUA Format/Requirements	Data Source and Limitations
Alaska	Began in 2020; expanded as additional tribal facilities have been onboarded	Line level for Tribal Health Organization facilities. No access for non-tribal facility data.	Tribal Health Organization facilities sign a DUA with the parent organization of the ANEC, granting the ANEC access to their data in the system. The state grants permission for line-level data for facilities with agreements. If state-level data are required, the ANEC works with the state to generate aggregate counts.	Data source: Facility-level, line-level data with PII, when the facilities have approved or requested it Limitation: This approach excludes data from non-tribal facilities, creating gaps in data surveillance for AIAN people who visit non-tribal facilities
South Dakota	December 2021	Aggregate	Aggregated data can be accessed due to the GPTEC’s status as a public health authority.	Data Source: All syndromic data reported in the South Dakota syndromic system Limitation: GPTEC can only access aggregated data without PII; IHS facilities do not submit data to this system
Idaho	2024	Line level	A confidentiality agreement restricts downloading line-level data and reporting data from single facilities.	Data Source: all syndromic data reported into the Idaho syndromic system Limitation: IHS facilities do not submit data to this system
Oregon	2019	Line level	A DUA restricts reporting or sharing data counts < 5. The state must approve a project proposal prior to analyzing and reporting data.	Data Source: All syndromic data reported in the Oregon syndromic system Limitation: IHS facilities do not submit data to this system
Washington	2017	Line level	A DUA restricts reporting or sharing data counts < 10. The state must approve all data communications before sharing with tribes.	Data Source: All syndromic data reported in the Washington State syndromic system Limitation: IHS facilities do not submit data to this system

Abbreviations: TEC, Tribal Epidemiology Center; DUA, data use agreement.

## Data Availability

No new data were created for this perspective study. The example reports were generated by the ANEC and the NWTEC using syndromic surveillance data to which they have access under their DUAs. Due to restrictions outlined in their DUAs, these data are not publicly available. However, the report created by the NWTEC can be accessed publicly through the following link: wtsc.wa.gov/wp-content/uploads/dlm_uploads/2024/02/2023-TR-4778_RHINO-Misclassification-of-AIAN.pdf (accessed on 14 April 2025).

## Abbreviations

The following abbreviations are used in this manuscript:

TEC	Tribal Epidemiology Center
AIAN	American Indian or Alaska Native
ANEC	Alaska Native Epidemiology Center
GPTEC	Great Plains Tribal Epidemiology Center
NWTEC	Northwest Tribal Epidemiology Center
NSSP	National Syndromic Surveillance Program
ED	Emergency Department
DUA	Data Use Agreement
PHA	Public Health Authority
IHCIA	Indian Health Care Improvement Act
HIPAA	Health Insurance Portability and Accountability Act
